# Comparison of the flexible parametric survival model and Cox model in estimating Markov transition probabilities using real-world data

**DOI:** 10.1371/journal.pone.0200807

**Published:** 2018-08-22

**Authors:** Xudong Du, Mier Li, Ping Zhu, Ju Wang, Lisha Hou, Jijie Li, Hongdao Meng, Muke Zhou, Cairong Zhu

**Affiliations:** 1 Department of Epidemiology and Biostatistics, West China School of Public Health, Sichuan University, Chengdu, Sichuan, China; 2 School of Aging Studies, University of South Florida, Tampa, Florida, United States of America; 3 Department of Neurology, West China Hospital, Sichuan University, Chengdu, Sichuan, China; The University of Hong Kong, CHINA

## Abstract

**Background and objective:**

Markov micro-simulation models are being increasingly used in health economic evaluations. An important feature of the Markov micro-simulation model is its ability to consider transition probabilities of heterogeneous subgroups with different risk profiles. A survival analysis is generally performed to accurately estimate the transition probabilities associated with the risk profiles. This study aimed to apply a flexible parametric survival model (FPSM) to estimate individual transition probabilities.

**Materials and methods:**

The data were obtained from a cohort study investigating ischemic stroke outcomes in Western China. In total, 585 subjects were included in the analysis. To explore the goodness of fit of the FPSM, we compared the estimated hazard ratios and baseline cumulative hazards, both of which are necessary to the calculate individual transition probabilities, and the Markov micro-simulation models constructed using the FPSM and Cox model to determine the validity of the two Markov micro-simulation models and cost-effectiveness results.

**Results:**

The flexible parametric proportional hazards model produced hazard ratio and baseline cumulative hazard estimates that were similar to those obtained using the Cox proportional hazards model. The simulated cumulative incidence of recurrent ischemic stroke and 5-years cost-effectiveness of Incremental cost-effectiveness Ratios (ICERs) were also similar using the two approaches. A discrepancy in the results was evident between the 5-years cost-effectiveness and the 10-years cost-effectiveness of ICERs, which were approximately 0.9 million (China Yuan) and 0.5 million (China Yuan), respectively.

**Conclusions:**

The flexible parametric survival model represents a good approach for estimating individual transition probabilities for a Markov micro-simulation model.

## Introduction

Markov Monte Carlo simulation models are being increasingly used in health economic evaluations[1–4]. The Markov Monte Carlo model can incorporate individual risk factors and an individual’s historical experiences (demographics and clinical history), and the effect of the risk factors is often reflected in the state-transition probabilities[5]. These transition probabilities are important input parameters in models used to inform clinical and policy decision-making [6–8]. Survival regression methods are commonly used to explore heterogeneity among patients and estimate the individual transition probability[9]. Traditional survival regression models can be divided as non-parametric, semi-parametric and parametric models. The next paragraph describes these models, the strengths and weaknesses.

Among the non-parametric survival models (i.e. the Kaplan-Meier method), a major limitation is that the influence of risk factors cannot be incorporated; thus, risk factors cannot be used to estimate the transition probabilities[10]. The Cox proportional hazard model is the most common model used in survival analyses[11]. However, the duration of the survey or trials is rarely sufficient to observe all events that occurred, and the survival function and cumulative hazards function are incomplete and cannot be extrapolated in Cox model; thus, the use of the Cox model in long-term health economic evaluation is limited[12]. Another disadvantage of the Cox proportional hazards model is that the survival function and cumulative hazards function of Cox model are step functions. However, it is reasonable to suppose that the underlying function is smooth[11]. Using the Cox model, a robust estimation of the transition probabilities may be achieved. This weakness may increase when estimating transition probabilities(i.e., the cumulative hazard may become zero in several time intervals). Alternatively, full-parametric survival models, such as the exponential and Weibull models, can be used to estimate transition probabilities. Using parametric survival models, we can estimate the smooth cumulative hazard functions and hazard ratios of the risk factors and extrapolate the survival functions and cumulative functions[13]. However, these models assume that the survival function and hazard function have a specific distribution. Thus, these models are usually not sufficiently flexible to be adequately representative, and the cumulative hazard function or survival function may be biased[11].

Although, the disadvantages of the abovementioned models are not unsurmountable, stratification may help alleviate the limitations of the non-parametric models, but the number of factors used for stratification is often limited[14, 15]. The Cox model is also useful and accurate if the sample size is large and the duration of the study or trial is sufficient[16]. Parametric survival models are reliable if the data obey the distribution assumptions[17]. However, these methods are frequently limited. In this study, we attempted to use the flexible parametric survival model (FPSM), which was developed by Royston and Parmar in 2001 and maybe a more reliable and universality method, to estimate transition probabilities. The FPSM is based on a series of models that are an extension of several standard survival models(i.e., Weibull, log-logistic)[18]. The FPSM has additional flexibility because the baseline distribution function is represented by a restricted cubic spline function of log time instead of simply a linear function of log time[19].

The purpose of this study was to apply the flexible parametric survival model to estimate individual transition probabilities and compared the Markov models constructed using FPSM with the Cox proportional hazards model. The validity and cost-effectiveness results of the two Markov models were compared.

## Materials and methods

### Flexible parametric survival analysis

The FPSM can be modeled on different scales, such as hazard scale, odd scale, or probit scale in flexible parametric survival analyses. In this study, we concentrate on models based on the hazard scale, which is an extension of the Weibull model and most commonly used. Thus, the regression results (hazards ratios) can be compared with the Cox model results. The Weibull model is a parametric proportional hazards model often criticized due to its lack of flexibility in the shape of the hazard function.

The log cumulative hazard function of a Weibull distribution can be written as follows:

$$\ln H(t;x)=\ln H_{0}(t)+x_{i}\beta_{i}=\lambda +\gamma \ln(t)+x_{i}\beta_{i}$$ (1)

ln*H*_0_(*t*) represents the baseline log cumulative hazard at time point *t*, *λ* is the scale parameter, *γ* is the shape parameter, *x*_*i*_ is the covariant and *β*_*i*_ is the parameter of the covariant.

Using a restricted cubic spline function of log time to transform the function[20], the log cumulative hazard function of the flexible parametric model based on a log cumulative hazard scale can be written as follows:

$$\ln H(t;x_{i})=\ln H_{0}\left(t\right)+\beta_{i}x_{i}=s\left(x\right)+\beta_{i}x_{i}$$ (2)

ln*H*_0_(*t*) represents the baseline log cumulative hazard at time point *t*, *x*_*i*_ is the covariant, *β*_*i*_ is the parameter of the covariant. *x* = ln(*t*), and *s*(*x*) is a restricted cubic spline function with *k* knots and parameters *γ*_0_…*γ*_*k*−1_ and can be written as follows:

$$s\left(x\right)=\gamma_{0}+\gamma_{1}z_{1}+\gamma_{2}z_{2}+\cdots +\gamma_{k-1}z_{k-1}$$ (3)

$$z_{1}=x=\ln(t)$$ (4)

where *z*_*j*_ (*j*≥2) are derived variables that are determined by the positions of knots. Details regarding the derived variables have been described by Royston and Parmar in their publication[11].

Modeling with spline functions requires the selection of the function complexity. The complexity is determined by the number and positions of connection points in log time, which are known as knots, of the spline's cubic polynomial segments[11]. If the number of knots is 0, the model degrades to the Weibull model, and *γ*_0_ and *γ*_1_ are equal to the scale parameter and shape parameter, respectively. Royston and Parmar suggest using 1 or 2 knots for smaller datasets and 4 or 5 for larger datasets (ten thousands of observations and more) as a reasonable choice[11].

The survival analysis was performed using Stata 12.0 (Stata-Corp LP, College Station, TX, USA). Additional details regarding the calculations of the transition probabilities are included in S1 Appendix.

### Data sources

To compare the Markov models constructed using the FPSM and Cox model, we developed a recurrent ischemic stroke Markov micro-simulation model as an example. We validated the two models by comparing the simulated cumulative incidence of recurrent ischemic stroke with the observed cumulative incidence of recurrent ischemic stroke in a cohort follow-up study. Then, we performed a cost-effectiveness analysis of antiplatelet therapy for secondary prevention of stroke and compared the cost-effectiveness results of the two models.

The main source of data in this study is a cohort follow-up study involving ischemic stroke survivors in China who constitute a subset of first-ever ischemic stroke patients hospitalized at the Department of Neurology, West China Hospital, Sichuan University. We administered a semi-structured questionnaire to collect information regarding the demographic characteristics, recurrent stroke and treatment, medication compliance, comorbidities, and quality of life (S1 Questionnaire). Trained interviewers conducted face-to-face interviews at the hospital and followed up every 3 months via telephone interviews after hospital discharge. In total, 775 first-ever ischemic stroke patients were recruited between January 2010 and June 2016, informed consent was obtained and the range of the follow up duration was 0 months to 75 months, until June 2016. In total, 51 cardioembolism patients were excluded because antiplatelet therapy was not suitable[21]. In addition, 68 patients were excluded because they only participated in the baseline interview only, and 71 patients were excluded because aspirin plus clopidogrel was indicated but is not appropriate for the long-term secondary prevention of stroke[21]. These 585 subjects were designated the original cohort and included in the analysis validating the Markov model. Information regarding all risk indicators was available for 570 of the 585 subjects. This group of subjects was designated the study cohort and included in the survival analysis. The study protocol was approved by the Medical Ethics Committee of West China Hospital, Sichuan University, Chengdu, China, with the following reference number: 200950.

### The Markov model

#### Model structure

**Fig 1:**
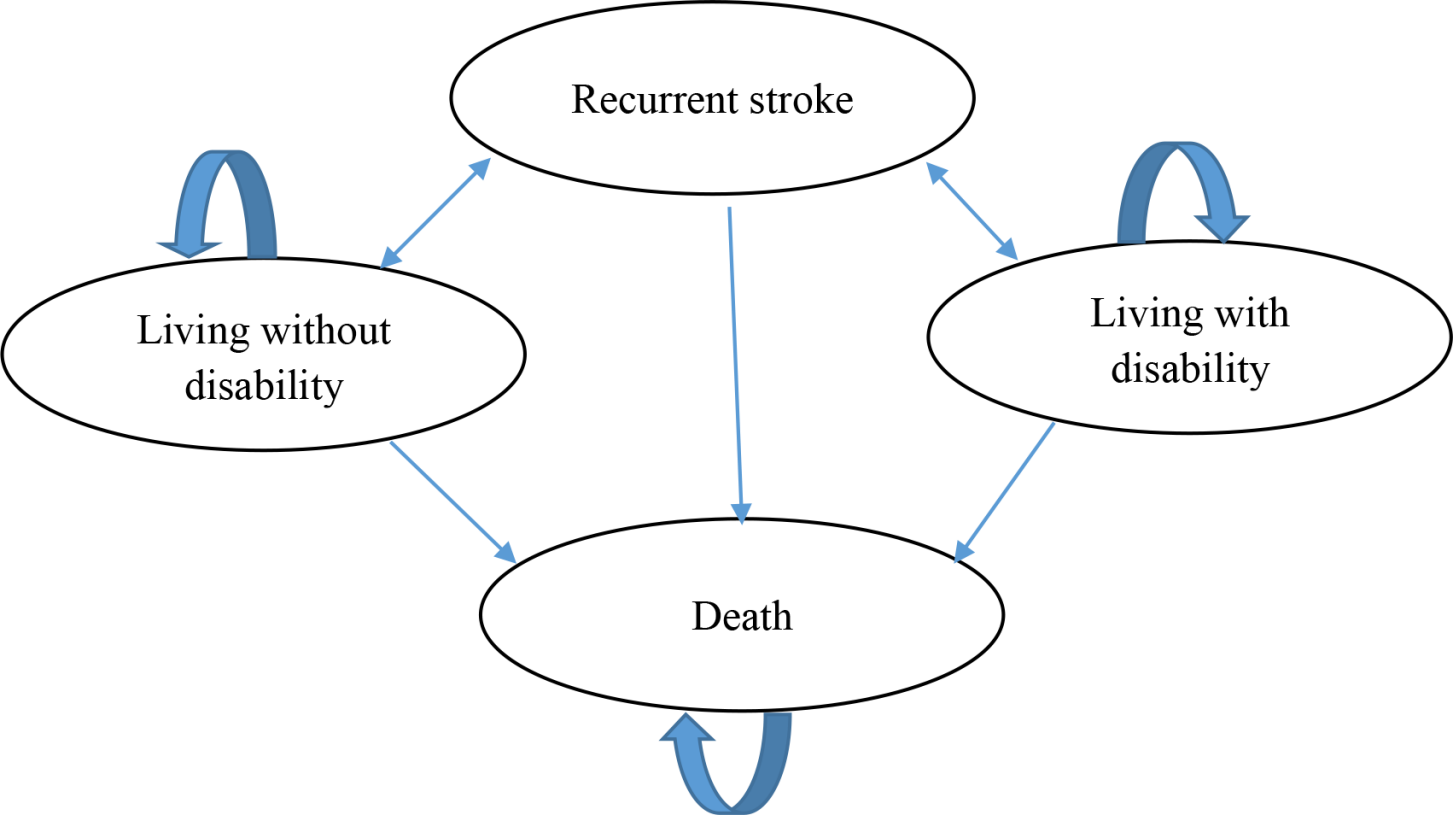
Simplified schematic of the recurrent stroke Markov micro-simulation model.

We built a recurrent ischemic stroke model using the Stroke Outcome Model (SOM) as a reference[22]. Our model is a state-transition model (schematically presented in Fig 1) including the following 4 states: 1) the death state, 2) the living with disability state, 3) the living without disability state, and 4) the recurrent stoke state; the recurrent stroke state is a temporary state. The cycle length is 3 months. During each cycle, the patients are at risk of having a fatal ischemic stroke event, a crippling ischemic stroke event, a non-fatal and non-crippling ischemic stroke event and non-ischemic stroke death event, or progression to the next cycle without suffering any event. The Markov model was built using TreeAge (version 2015, TreeAge Software Inc., Williamstown, MA) and we set random seed as 1. The key parameters used in the analysis are summarized in Table 1. The Markov micro-simulation model structure in TreeAge Pro is presented in S1 Fig.

**Table 1:** Key parameters used in the Markov model.

Parameter	Base-Case	Uncertainty Range and Distribution	Sources
**Efficacy of antiplatelet therapy (Hazard ratio)**			This study
**Aspirin**	0.78	0.3597–1.6799, Normal	
**Clopidogrel**	0.65	0.2900–1.4525, Normal	
**Daily cost (CNY)**			NDRC[23]
**Aspirin (100 mg/day)**	0.50	0.40–0.60, Normal	
**Clopidogrel (75 mg/day)**	19.57	15.66–23.49, Normal	
**Unit event cost of ischemic stroke(CNY)**^a^	8777	±25%,uniform)	Ming et al [24]
**Utilities of ischemic stroke**			This study
Non-disabled	0.80	±25%, uniform	
Disabled	0.59	±25%, uniform	
Discounting	3%	0–8%, uniform	Liu et al[25]
Event probabilities			This study
Death after ischemic stroke	0.2540	0.1769–0.3310, Normal	
Disability after ischemic stroke	0.3111	0.2735–0.3487, Normal	

^a^ Unit event costs of ischemic stroke include all medical costs and medication costs during hospitalization, CNY = China Yuan

#### Event probabilities

To estimate the individual probabilities of recurrent stroke with different risk indicator patterns, we constructed an ischemic recurrent stroke probability function based on a survival analysis. The recurrent ischemic stroke risk factors considered in this study included age, heart disease, systolic blood pressure, modified Rankin score (MRS), antiplatelet therapy, and medication adherence. Large studies have previously identified these risk factors[21]. Systolic blood pressure was obtained from follow-up data as the average level during the follow-up time and classified as <140 mmHg or ≥140 mmHg. The MRS, which reflects the severity of the first-ever ischemic stroke, was measured using Modified Rankin Scale, where 0–2 is defined as non-disability and 3–5 is defined as disability. Antiplatelet therapy included aspirin(100mg/d), clopidogrel(75mg/d), or no antiplatelet therapy. Medication adherence refers to adherence to antiplatelet therapy and was defined by the proportion of days covered (PDC) and classified as ≥0.8 or <0.8[26].

The age- and sex-specific risk of non-ischemic stroke death events were set based on the *China Health Statistics Yearbook 2015*(S1 Table)[27]. We set the disability probability after ischemic stroke to 0.3111, which is the proportion of disabled patients in our original cohort. The fatality probability after stroke is 0.254, for 126 patients in the original cohort suffered from recurrent ischemic stroke events, and 32 of these patients died.

### Validity of the Markov models

To compare the validity of the model constructed based on the FPSM and that of the Cox model, we studied how closely the simulated cumulative incidence of recurrent ischemic stroke agreed with the observed cumulative incidence of recurrent ischemic stroke. To simulate the mean and distribution of the outcomes, we performed 100 2nd-order Monte Carlo simulations, and with each simulation, consecutively simulated the number of subjects in the original cohort (100 × 585).

### Utility and cost

To compare the cost-effectiveness results of these two model, additional parameters were necessary. The utility values of living without disability state, living with disability state and death state were 0.80, 0.59 and 0, respectively, as measured by the SF-6D instrument. The details of the utility measurements among this cohort of ischemic stroke survivors have been described in another paper [28]. The cost estimates include aspirin and clopidogrel medication cost, and all medical costs and medication costs during hospitalization if an ischemic stroke event occurred. The medication costs for clopidogrel and aspirin were calculated using unit costs obtained from the highest prices published by the National Development and Reform Commission (NDRC) based on a daily dose of 75 mg for clopidogrel and 100 mg for aspirin [23]. The costs and utilities were discounted at 3%/y.

## Results

### Study population

During the follow-up, 124 recurrent stroke events, 30 stroke deaths, and 55 non-stroke deaths occurred in the study cohort, while 126 recurrent stroke events, 32 stroke deaths, and 59 non-stroke deaths, occurred in the original cohort. The baseline characteristics of the study cohort and original cohort are described in Table 2. The characteristics of these two groups were highly comparable.

**Table 2:** Characteristics of the study cohort and original cohort.

Characteristics	Study cohort (N = 570)	Original cohort (N = 585)
**Male sex, N (%)**	349(61.23)	355(60.79)
**Age, mean ±standard deviation**	62.01±12.66	62.05±12.65
**Presence of heart disease, N (%)**	106(18.60)	111(18.97)
**Systolic blood pressure** ≥140**mm Hg, N (%)**	212(37.19)	214(36.83)
**MRS** ≥3, **N (%)**	175(30.70)	182(31.11)
**Antiplatelet therapy, N (%)**		
Aspirin	346(60.70)	352(60.17)
Clopidogrel	179(31.40)	185(31.62)
None	45(7.89)	48(8.21)
**PDC ≥0.80, N (%)**	304(53.33)	322(55.04)

### Survival analysis

Both the Cox model and flexible parametric model were fitted to compare the two models in terms of the recurrent ischemic stroke hazard ratios and baseline cumulative hazards. As shown in Table 3, the hazard ratios estimated by the Cox Model and flexible parametric model are very similar. Age, heart disease, systolic blood pressure, MRS, and medication adherence are significant risk factors for recurrent ischemic stroke.

**Table 3:** Hazard ratios (95% confidence intervals) of the flexible parametric survival model (FPSM) and Cox proportional hazards model (Cox).

Variables	Recurrent stroke	
	FPSM	Cox
**Age**	1.0289(1.0126,1.0455)	1.0289(1.0126,1.0454)
**Heart disease (referent: no heart disease)**	1.7312(1.1527,2.6000)	1.7039(1.1341,2.5601)
**Systolic blood pressure**≥140**mmHg (referent: <140 mmHg)**	1.9077(1.3298,2.7368)	1.8674(1.3016,2.6791)
**MRS ≥ 3 (referent: < 3)**	1.6985(1.1803,2.4442)	1.6787(1.1656,2.4177)
**Antiplatelet therapy (referent: no therapy)**	-	-
Aspirin	0.7773(0.3597,1.6799)	0.7851(0.3633,1.6968)
Clopidogrel	0.6491(0.2900,1.4525)	0.6625(0.2959,1.4829)
**Medication adherence: PDC <0.8 (referent: PDC≥0.80)**	1.9225(1.3118,2.8175)	1.8569(1.2656,2.7244)

**Fig 2:**
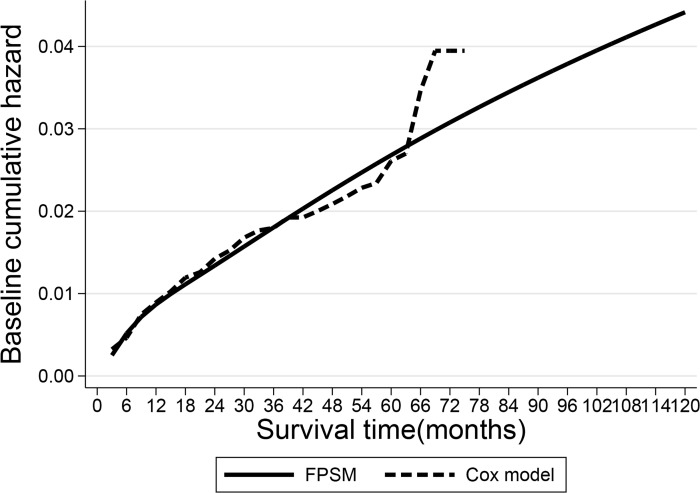
Comparison of the baseline cumulative hazards of the Cox model and flexible parametric survival model.

Fig 2 shows the estimates of the baseline cumulative hazard from the Cox model and flexible parametric survival model. The results of the two approaches were similar. The flexible parametric model provides smooth estimates of the baseline cumulative hazards extrapolated to 10 years. The Cox model provides an unsmooth curve that ends at the final follow-up time point. The baseline cumulative hazard estimates by Cox model exhibit an unusual increase in the 6th year. This increase was likely due to the few patients completing the 6 years of follow-up in our study, and thus, this estimator may be unreliable. The specific value of the baseline cumulative hazard is shown in S2 Table.

### Validity of the Markov models

**Fig 3:**
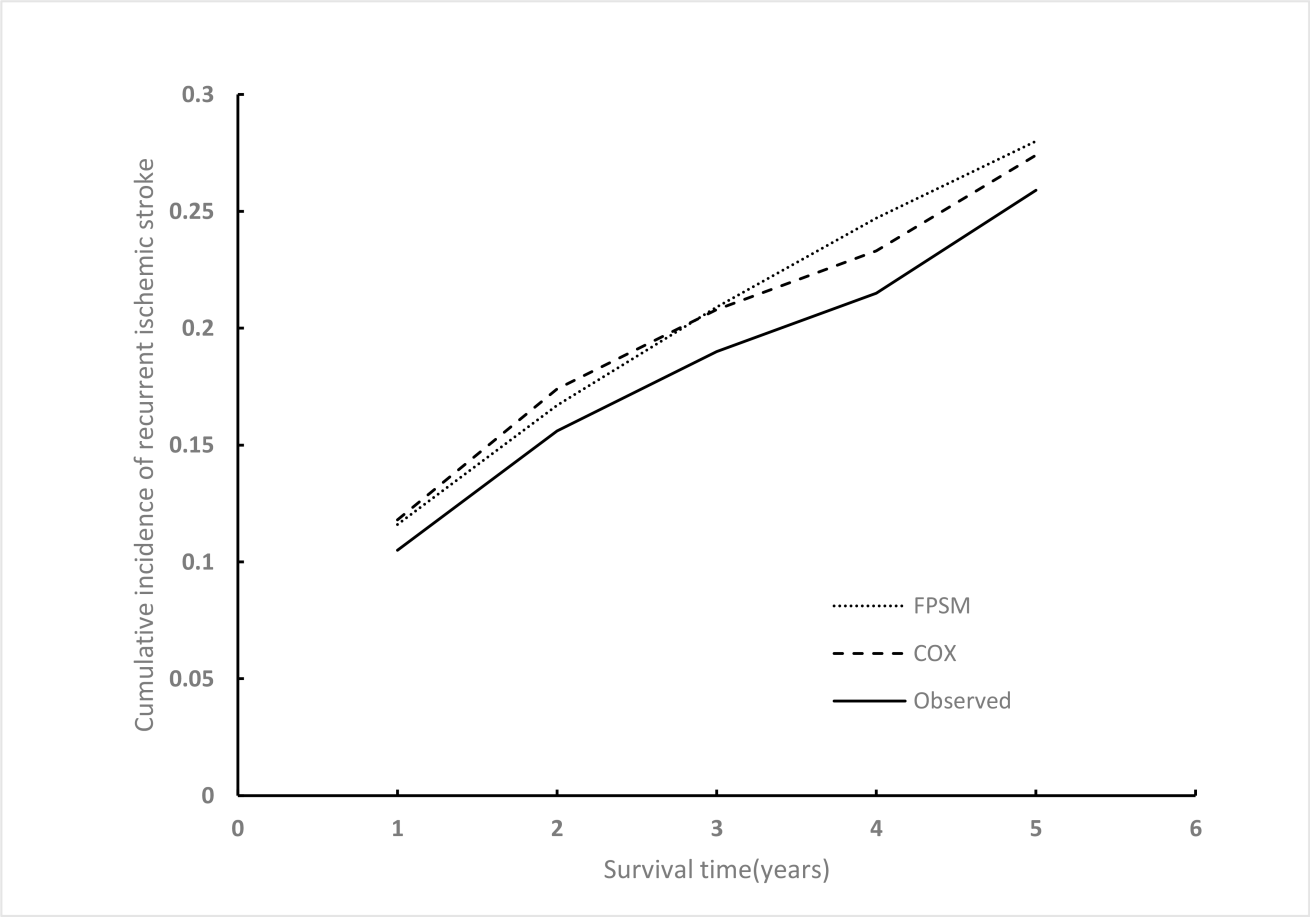
Cumulative incidence of recurrent ischemic stroke over 5 years of follow-up.

The Kaplan-Meier estimates of the observed cumulative incidence of recurrent ischemic stroke were 0.105, 0.156, 0.190, 0.215, and 0.259, at 1 year, 2 years, 3 years, 4 years, and 5 years, respectively, in the original cohort. Fig 3 shows the cumulative incidence of recurrent ischemic stroke over the 5 years of follow-up estimated using the three methods (observed in the original cohort, and estimated using Monte Carlo–Markov simulation model using a Cox model and a flexible parametric model. The two model approaches were very close but slightly overestimate the cumulative incidence. The distribution of the simulated cumulative incidence event is shown in S2 Fig.

### Cost- effectiveness analysis results

The results of the cost-effectiveness analysis are summarized in Table 4. The 5-year cost-effectiveness results were very similar between the FPSM and Cox model both in the QALYs and cost. Compared with aspirin, patients receiving clopidogrel had higher medical costs, but the QALYs gained were higher, while the patients without antiplatelet therapy had higher medical costs and lower QALYs. The incremental cost-effectiveness ratio (ICER) of clopidogrel to ASA was approximately 1 million CNY. Since the survival function or cumulative hazard function can be extrapolated by the FPSM, we simulated the 10-year cost-effectiveness, and the results showed that the use of clopidogrel still had higher costs, and higher QALYs gained compared with aspirin, but the ICER changed considerably, and may significantly influencing the final decision.

**Table 4:** Incremental Cost-effectiveness Ratios.

	FPSM			Cox model		
	No	Aspirin	Clopidogrel	No	Aspirin	Clopidogrel
**5 years**						
**Mean cost (CNY)**	3318	3199	24241	3220	3104	24155
**Mean QALYs**	3.013	3.058	3.082	3.023	3.063	3.086
**Cost per QALY gained (CNY)**	-2644	-	876750	-2900	-	915260
**10 years**	4986	4933	40960			
**Mean cost (CNY)**	4986	4933	40960			
**Mean QALYs**	5.068	5.188	5.263			
**Cost per QALY gained (CNY)**	-442	-	480360			

CNY = China Yuan; QALY = quality-adjusted life year

### Sensitivity analysis

Probabilistic sensitivity analyses (PSA) were performed to assess the impact of the uncertainty of the model parameters (i.e., hazard ratios of risk factors, discount, and proportions of different patient characteristics) varying simultaneously with the distribution. The distribution of the hazard ratios of the risk factors were lognormal, the distribution of the proportions of the different patient characteristics were considered normal, and the distributions of the other parameters are defined in Table 1. The willingness-to-pay (WTP) threshold per QALY gained is defined as three times the gross domestic product (GDP) per capita (53980*3 CNY = 161940 CNY) according to the World Health Organization recommendation[29]. GDP per capita in China was obtained from the *China Statistics Yearbook 2017*[30].

The results of the PSA of 5-year cost-effectiveness were similar using the Cox model and FPSM, and 10% of the ICERs of clopidogrel were lower than the WTP (161940 CNY). The result of PSA of 10-year cost-effectiveness using the FPSM differed from the 5-year cost-effectiveness results, and 16% of the ICERs of clopidogrel were lower than the WTP (161940 CNY). The cost-effectiveness acceptability curves are shown in S3 Fig.

## Discussion

The flexible parametric survival model has been widely used in many areas, such as relative survival and clinical decisions[31], but few studies have used the model in economic evaluations of health-care technologies. In this article, we applied the flexible parametric survival model to estimate Markov individual transition probabilities and compared the simulation results of the cumulative incidence of recurrent ischemic stroke, QALYs and cost-effectiveness between a Cox proportional hazards model and a flexible parametric proportional hazards model.

The flexible parametric model is an alternative way to estimate transition probabilities. This approach can directly use the individual patient data to model the transitions rather than using a priori transition probabilities that are often used in a Markov cohort model, and can allow transition probabilities to vary across different patients. This approach also allows the transition probabilities to vary over time as the baseline cumulative hazards change over time. Using individual patient data and incorporating additional covariate information could be worthwhile and lead to improved predictions of transition probabilities with reduced uncertainty[13].

A parametric survival model with a survival function fitted to empirical survival data requires assumptions concerning the shape of the distribution and degree of error between the fitted and empirical data. The flexible parametric survival model may remit the error. To prove this, we compared FPSM with Cox proportional hazards model, it is acknowledged that Cox model performed well in fitting survival data. In our study, the FPSM produces estimates that are very similar to those produced by the Cox proportional hazards model in terms of the hazard ratios of the risk factors. An additional comparison between the baseline cumulative hazard was performed. The Cox proportional hazards model obtained the baseline cumulative hazard using the Breslow method[32], and the hazards were only defined at time-points during which events occurred. In our analysis, the baseline cumulative hazard during certain intervals after the first-ever ischemic stroke were 0 (i.e., 39 months to 42 months) because no events occurred during this period; thus, the baseline cumulative hazards appear unreliable, while the FPSM provided reliable and smooth estimates of the baseline cumulative hazards[31]. However, the simulation results of the cumulative incidence of recurrent ischemic stroke were also similar using the two approaches (Fig 3); their distinction had only a slight influence in this study, and we prudently considered the FPSM to be more reliable.

Notably, the hazard ratio of antiplatelet drugs was not significant, likely because the antiplatelet drug choices doctors make are appropriate for each patient considering the high medical level of West China Hospital. Since many studies have identified the difference between aspirin and clopidogrel[33], we argue that the directions of the hazard ratio is consistent with other studies, and should be used as a best estimate of the effect for estimated transition probabilities, rather than simply assuming no effect, which is likely due to the lack of statistical power, and to perform a cost-effectiveness analysis, this risk factor should also be used. The uncertainty can be addressed by performing sensitivity analyses.

As shown in Fig 3, the simulation results of the two model approaches were similar, but the models appeared to slightly overestimated the cumulative incidence of recurrent ischemic stroke for the observed cumulative incidence. The basis may due to other parameters in Markov model. For example, the stroke survivors have worse health condition than general population, the risk of non-ischemic stroke death events from *China Health Statistics Yearbook 2015* was underestimate. However, the mean difference of 5 years was approximately 0.015, which may be receivable.

Notably, the flexible parametric model has another advantage that it can be extrapolated to unobserved period, whereas the Cox model is limited. The different time horizon assumptions used for the modeling of the transitions may lead to different results. In particular, the difference lead to differing ICERs, which could affect the conclusions regarding cost-effectiveness analysis. The 5-year cost-effectiveness results differed from the 10-year cost-effectiveness results in our study. Thus, extrapolation of survival is needed to provide more information regarding cost-effectiveness to decision makers.

This study focused on the validity of using a flexible parametric model to estimate individual transition probabilities; thus, the cost-effectiveness analysis in this study appears slightly robust. For example, we did not consider the bleeding events, which are a common side effect of aspirin and may influence the cost-effectiveness results. Considering that the major advantages of the current Markov micro-simulation model are that the model can incorporate individual risk factor profiles and memory of the individual life histories and allows the transition probabilities to change in response to the life histories over the simulation, our evaluation was not realistic. In our study, we incorporate few risk factors and only consider age a variational risk factor and fixed the others factors, but certain factors, such as medication adherence, may change over time[4], and interactions between risk factors were not considered in this study; further studies can improve the Markov model to produce a more accurate cost-effectiveness evaluation.

In summary, using the Markov micro-simulation model, we present “real world” probabilities in which patients have different characteristics, and the flexible parametric model provides an approach to estimating individual transition probabilities in a Markov micro-simulation model. The flexible parametric model produced results that were similar to those produced using the Cox model. Considering the excellent good-ness of fit of the flexible parametric survival model and the importance of extrapolating survival in long-term cost-effectiveness analysis, the flexible parametric model provides a good approach to estimating individual transition probabilities in Markov micro-simulation models used for health economics evaluation.

## Supporting information

S1 Appendix Calculate the individual transition probabilities using a flexible parametric proportional hazards model. (DOCX)

S1 Table Sex-age- death probabilities (3 months). (DOCX)

S2 Table Baseline cumulative hazard estimated using the FPSM and Cox model. (DOCX)

S1 Fig Structure of the Markov micro-simulation model. (TIF)

S2 Fig Distribution of the simulated cumulative incidence of recurrent ischemic stroke. (TIF)

S3 Fig Cost-effectiveness acceptability curve. (TIF)

S1 Dataset Original cohort data and study cohort data. (RAR)

S1 Questionnaire The survey questions used in the study. (RAR)
