# Supplementary material for: Comparison of the flexible parametric survival model and Cox model in estimating Markov transition probabilities using real-world data
Source: PLoS One. 2018 Aug 22;13(8):e0200807. doi: 10.1371/journal.pone.0200807 (PMC6104919; doi:10.1371/journal.pone.0200807)
Supplement: S1 Appendix — (DOCX) [file pone.0200807.s001.docx]

**S1 Appendix. Calculate** **the individual transition probabilities using a flexible parametric proportional hazards model**

1) Define the length of the Markov cycle as *u* and let the cumulative hazard of the event(recurrent ischemic stroke at time *t* be represented by *H*(*t*). 2) Define *tp*(*t_u_*) as the discrete transition probability between time-points *t*－*u* and *t*. 3) The transition probability of the event of interest can simply be defined as one minus the ratio of the survivor function at the end of the interval to the survivor function at the beginning of the interval as follows:

$tp(t_{u})=1-S(t;x)/S(t-u;x)$ (1)

This equation can be rewritten in terms of the cumulative hazard as follows:

$$tp(t_{u})=1-\exp\{-H(t;x)\}/\exp\{-H(t-u;x)\}$$

$$\text{=}1-\exp\{H(t-u;x)-H(t;x)\}=1-\exp\{H(t-u;x)-H(t;x)\}$$

$\text{=}1-\exp\{[\exp(\beta_{i}x_{i})][H_{0}(t-u)-H_{0}(t)]\}$ (2)

where $x_{i}$ is a risk factor and$\beta_{i}$ is the coefficient of the risk factor.

Therefore, the most important step in the calculation of the transition probabilities is the estimation of log cumulative hazards function of the flexible parametric model based on a log cumulative hazard scale as follows:

(3)

$z_{j}$ is the derived variable and we have $z_{1}\text{=}x=\ln(t)$, and *j*=2,…,*m*+1,

(4)

and $\lambda_{j} =\frac{k_{\max} -k_{j} }{k_{\max} -k_{\min} }$ (5)

where and are the smallest and largest uncensored event times in the data, is the uncensored event times of the *j*th interior knot, and .

In our study, the number of interior knots is *m*=2, and the positions of the internal knots are on the 33rd and 67th centiles of the uncensored event times, which is the default in Stata software. The smallest and largest uncensored event times are 1.5 and 67.5months; thus, the and , and the 33rd and 67th centiles of the uncensored event-time are 7.5 and 25.5 months; thus, $k_{2}=2.0149$and$k_{3}=3.2387$. Therefore, we find that and .

Consider a 50-year-old man, with heart disease, SBP ≥140 mmHg, and MRS≥3, who received aspirin and had good medication adherence (PDC ≥0.8). We estimate the transition probability from living with disabled state to entering a recurrent ischemic stroke state between time-points 3 months and 6 months.

Step 1: Using formula (4) to calculate the derived variables $z_{j}$ at 3 months and 6 months.

3 months: $z_{1}\text{=}x=\ln(t)\text{=}\ln(3)$

6 months: $z_{1}\text{=}x=\ln(t)\text{=}\ln(6)$

Step 2: Calculating the baseline cumulative hazards at 3 and 6 months.

The coefficients of the risk factors and derived variables are listed in Table A.

Baseline cumulative hazards at 3 months=

=exp (-7.3172+1.2284*ln (3) + 0.1839*-0.1922-0.1970*-0.0852) =0.002513, and the baseline cumulative hazards at 6 months=0.005171.

Step 3: Using formula (2) to calculate the transition probability

The transition probability between time-points 3 months and 6 months=1-exp[(0.002513- 0.005171)*exp(50*0.0285+0.5488*1+0.6459*1+0.5297*1-0.2519*1+0.6536*0)]=0.047044.

Notably, we can easily obtain the baseline cumulative hazards using the Stata software stpm2 module, and we generally do not need to perform many calculations.

Table A. The coefficients of the risk factors and derived variables.

| **Variables** | coefficient |
| --- | --- |
| **Age** | 0.0285 |
| **Heart disease (referent: no heart disease)** | 0.5488 |
| **Systolic blood pressure**≥140**mm Hg (referent: <140)** | 0.6459 |
| **MRS ≥ 3 (referent: < 3)** | 0.5297 |
| **Antiplatelet therapy (referent: no therary)** | - |
| aspirin | -0.2519 |
| clopidogrel | -0.4322 |
| **Medication adherence: PDC<0.8 (referent: PDC≥0.80)** | 0.6536 |
| $\gamma_{0}$ | -7.3172 |
| $\gamma_{1}$ | 1.2284 |
| $\gamma_{2}$ | 0.1839 |
| $\gamma_{3}$ | -0.1970 |
