# Supplementary material for: Comparison of the flexible parametric survival model and Cox model in estimating Markov transition probabilities using real-world data
Source: PLoS One. 2018 Aug 22;13(8):e0200807. doi: 10.1371/journal.pone.0200807 (PMC6104919; doi:10.1371/journal.pone.0200807)
Supplement: S1 Table — (DOCX) [file pone.0200807.s002.docx]

S1 Table. Sex-age- death probabilities (3-months)*

| Age | Male | Female |
| --- | --- | --- |
| 0~ | 0.001493 | 0.001104 |
| 1~4 | 0.000124 | 0.000097 |
| 5~9 | 0.000058 | 0.000037 |
| 10~14 | 0.000065 | 0.000042 |
| 15~19 | 0.000095 | 0.000037 |
| 20~24 | 0.000093 | 0.000039 |
| 25~29 | 0.000154 | 0.000070 |
| 30~34 | 0.000211 | 0.000093 |
| 35~39 | 0.000269 | 0.000116 |
| 40~44 | 0.000521 | 0.000220 |
| 45~49 | 0.000646 | 0.000278 |
| 50~54 | 0.001365 | 0.000598 |
| 55~59 | 0.001712 | 0.000726 |
| 60~64 | 0.003074 | 0.001512 |
| 65~69 | 0.004938 | 0.002655 |
| 70~74 | 0.007525 | 0.004351 |
| 75~79 | 0.011928 | 0.008110 |
| 80~84 | 0.021972 | 0.016312 |
| 85~ | 0.047841 | 0.039780 |

* The annual death probabilities was changed to 3-months death probabilities by using conversion formula: $P=1-e^{-rt}$
