# Supplementary material for: Comparison of the flexible parametric survival model and Cox model in estimating Markov transition probabilities using real-world data
Source: PLoS One. 2018 Aug 22;13(8):e0200807. doi: 10.1371/journal.pone.0200807 (PMC6104919; doi:10.1371/journal.pone.0200807)
Supplement: S2 Table — (DOCX) [file pone.0200807.s003.docx]

S2 Table. The baseline cumulative hazard estimated by FPSM and Cox model

| **Time (month)** | Baseline cumulative hazard | | **Time (month)** | Baseline cumulative hazard | |
| --- | --- | --- | --- | --- | --- |
|  | FPSM | Cox model |  | FPSM | Cox model |
| 3 | 0.002513 | 0.003237 | 63 | 0.027821 | 0.026992 |
| 6 | 0.005171 | 0.004647 | 66 | 0.028823 | 0.034606 |
| 9 | 0.007172 | 0.007470 | 69 | 0.029805 | 0.039470 |
| 12 | 0.008679 | 0.008929 | 72 | 0.030769 | 0.039470 |
| 15 | 0.009952 | 0.010267 | 75 | 0.031715 | 0.039470 |
| 18 | 0.011123 | 0.011937 | 78 | 0.032643 | - |
| 21 | 0.012260 | 0.012602 | 81 | 0.033555 | - |
| 24 | 0.013402 | 0.014234 | 84 | 0.034450 | - |
| 27 | 0.014557 | 0.015222 | 87 | 0.035331 | - |
| 30 | 0.015716 | 0.016794 | 90 | 0.036197 | - |
| 33 | 0.016875 | 0.017670 | 93 | 0.037048 | - |
| 36 | 0.018029 | 0.017972 | 96 | 0.037886 | - |
| 39 | 0.019175 | 0.019244 | 99 | 0.038710 | - |
| 42 | 0.020309 | 0.019244 | 102 | 0.039522 | - |
| 45 | 0.021430 | 0.020014 | 105 | 0.040322 | - |
| 48 | 0.022538 | 0.020868 | 108 | 0.041109 | - |
| 51 | 0.023629 | 0.021811 | 111 | 0.041885 | - |
| 54 | 0.024704 | 0.022838 | 114 | 0.042650 | - |
| 57 | 0.025761 | 0.023422 | 117 | 0.043404 | - |
| 60 | 0.026800 | 0.026100 | 120 | 0.044148 | - |
