# Supplementary figures and images for: Comparison of the flexible parametric survival model and Cox model in estimating Markov transition probabilities using real-world data
Source: PLoS One. 2018 Aug 22;13(8):e0200807. doi: 10.1371/journal.pone.0200807 (PMC6104919; doi:10.1371/journal.pone.0200807)

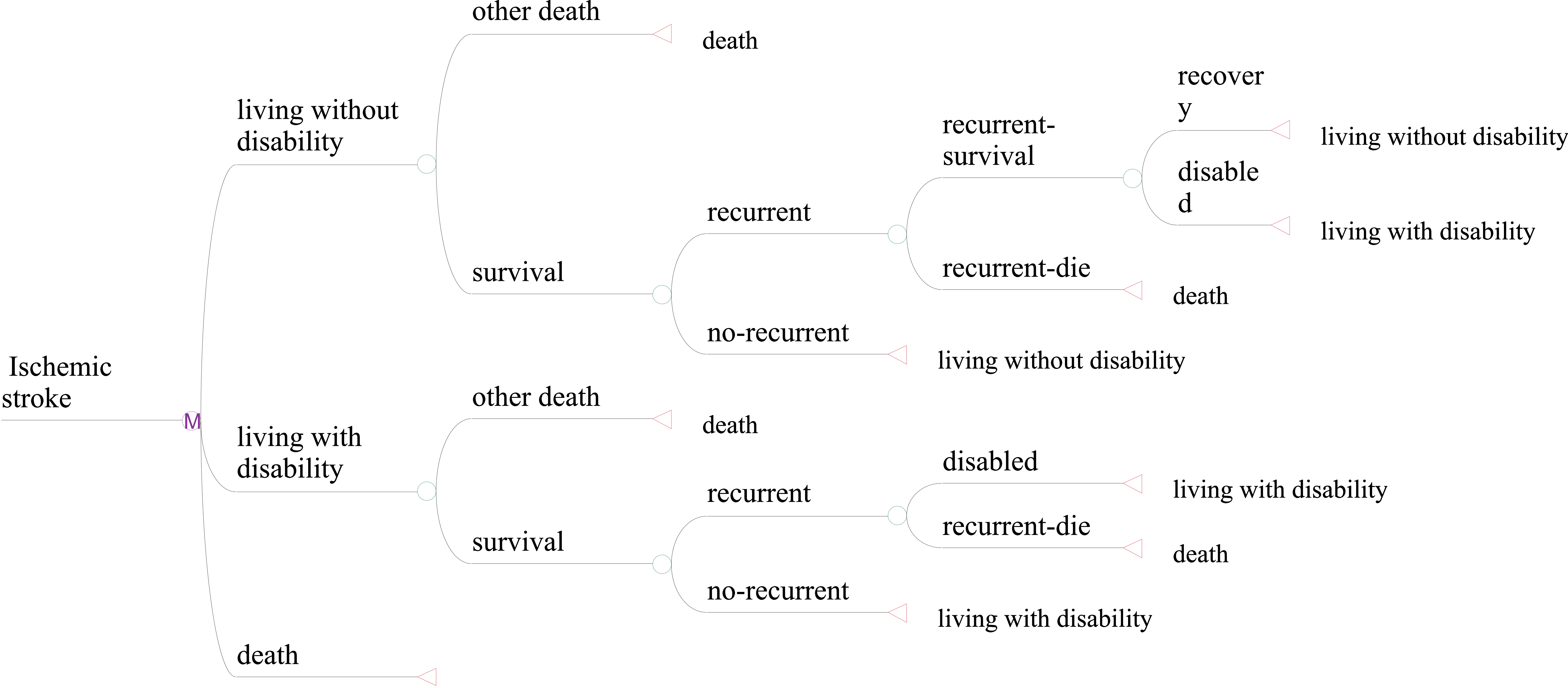

Supplement: S1 Fig — (TIF) [file pone.0200807.s004.tif]
